# Sphingolipid metabolism potential in fecal microbiome and bronchiolitis in infants: a case–control study

**DOI:** 10.1186/s13104-017-2659-9

**Published:** 2017-07-26

**Authors:** Kohei Hasegawa, Christopher J. Stewart, Jonathan M. Mansbach, Rachel W. Linnemann, Nadim J. Ajami, Joseph F. Petrosino, Carlos A. Camargo

**Affiliations:** 1 Department of Emergency Medicine, Massachusetts General Hospital, Harvard Medical School, 125 Nashua Street, Suite 920, Boston, MA 02114-1101 USA; 2 Department of Molecular Virology and Microbiology, Alkek Center for Metagenomics and Microbiome Research, Baylor College of Medicine, Houston, TX USA; 3 Department of Medicine, Boston Children’s Hospital, Boston, MA USA; 4 Department of Pediatrics, Emory University School of Medicine, Atlanta, GA USA

**Keywords:** Microbiome, Infants, Bronchiolitis, *Bacteroides*, Sphingolipids

## Abstract

**Objective:**

Emerging evidence demonstrated that the structure of fecal microbiome is associated with the likelihood of bronchiolitis in infants. However, no study has examined functional profiles of fecal microbiome in infants with bronchiolitis. In this context, we conducted a case–control study. As a part of multicenter prospective study, we collected stool samples from 40 infants hospitalized with bronchiolitis (cases). We concurrently enrolled 115 age-matched healthy controls.

**Results:**

First, by applying 16S rRNA gene sequencing to these 155 fecal samples, we identified the taxonomic profiles of fecal microbiome. Next, based on the taxonomy data, we inferred the functional capabilities of fecal microbiome and tested for differences in the functional capabilities between cases and controls. Overall, the median age was 3 months and 45% were female. Among 274 metabolic pathways surveyed, there were significant differences between bronchiolitis cases and healthy controls for 37 pathways, including lipid metabolic pathways (false discovery rate [FDR] <0.05). Particularly, the fecal microbiome of bronchiolitis cases had *consistently* higher abundances of gene function related to the sphingolipid metabolic pathways compared to that of controls (FDR <0.05). These pathways were more abundant in infants with *Bacteroides*-dominant microbiome profile compared to the others (FDR <0.001). On the basis of the predicted metagenome in this case–control study, we found significant differences in the functional potential of fecal microbiome between infants with bronchiolitis and healthy controls. Although causal inferences remain premature, our data suggest a potential link between the bacteria-derived metabolites, modulations of host immune response, and development of bronchiolitis.

## Introduction

Bronchiolitis is a common acute respiratory infection and the leading cause of hospitalizations in US infants [1, 2]. Although bronchiolitis has been considered virus-induced inflammation of small airways [3], recent studies demonstrate that the pathobiology involves complex interrelations among respiratory viruses, host immune response, and human microbiome [4–10]. Emerging evidence also indicates the existence of “gut-lung axis” in which the gut microbiome conditions immunologic responses in the lungs to environmental challenges (e.g., viral infection) [11]. Indeed, we have previously demonstrated, in a case–control study of infants hospitalized for bronchiolitis and healthy controls [11], that the taxonomy profiles of the fecal microbiome were associated with the likelihood of bronchiolitis—e.g., infants with the *Bacteroides*-dominant profile were more likely to have bronchiolitis. Although previous studies suggest that the gut microbiome-derived metabolites (e.g., sphingolipids) may play an important role in the host immune development [12, 13], the functional profiles of fecal microbiome in infants were not examined in the earlier study. To address this knowledge gap, we determined the predicted function of fecal microbiome in infants with bronchiolitis and healthy infants.

## Main text

### Methods

This study was a secondary analysis of the data from a case–control study of infants hospitalized for bronchiolitis and healthy controls. The study design, setting, participants, and methods of data collection have been reported previously [11]. In brief, as a part of a multicenter prospective cohort study, called the 35th Multicenter Airway Research Collaboration (MARC-35) [4–7, 9], we enrolled 40 infants (aged <12 months) hospitalized for an attending physician diagnosis of bronchiolitis from November 2013 through April 2014. Bronchiolitis was diagnosed according to the American Academy of Pediatrics guidelines [14]. Exclusion criteria were a transfer to a participating hospital >48 h after the original hospitalization, delayed consent (>24 h after hospitalization), gestational age ≤32 weeks, and known comorbidities (cardiopulmonary disease, immunodeficiency, immunosuppression). In addition, during the same period, we also enrolled 115 healthy infants as the controls (age-matched within 1.5 months of cases) [11, 15–17]. We excluded infants with current fever, respiratory illness, or gastrointestinal illness, antibiotic treatment in the preceding 7 days, gestational age ≤32 weeks, or known comorbidities. Taken together, a total of 155 infants were eligible for the current analysis. From these infants, by using a standardized protocol [11, 15, 17], investigators conducted a structured interview and medical record review, and collected fecal specimens at the time of hospitalization (cases) or at home before the clinic visit (controls). The fecal samples were immediately stored at −80 °C. The institutional review board at each of the participating hospitals approved the study. Written informed consent was obtained from the parent or guardian.

**Fig. 1:**
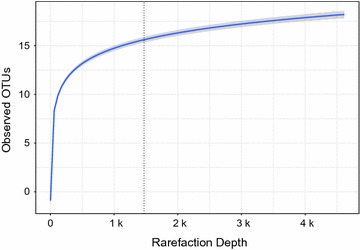
Rarefaction curves for bacterial operational taxonomic units of the fecal microbiome. The *horizontal axis* indicates sequence depth while the *vertical axis* indicates the number of bacterial operational taxonomic units (OTUs). All 155 fecal specimens had sufficient depth to obtain high degree of sequence coverage (rarefaction cutoff, 1470 reads/specimen)

16S rRNA gene sequencing was performed based on the methods adapted from the NIH Human Microbiome Project. Briefly, bacterial genomic DNA was extracted using MO BIO PowerMag DNA Isolation Kit (Mo Bio Lab; Carlsbad, CA). The 16S rDNA V4 regions were amplified by PCR and sequenced in the MiSeq platform (Illumina; SanDiego, CA) using 2 × 250 bp paired-end protocol. Sequencing read pairs were demultiplexed based on the unique molecular barcodes, and reads were merged using USEARCH v7.0.1090, allowing no mismatches and a minimum overlap of 50 bases. We trimmed the merged reads at the first base with a Q5 quality score. We calculated the expected error after taking into account all Q scores across all the bases of a read and the probability of an error occurring. We also applied a quality filter to the resulting merged reads, discarded the reads containing >0.05 expected errors. We constructed rarefaction curves of bacterial operational taxonomic units (OTUs) using sequence data for each sample to ensure coverage of the bacterial diversity present (Fig. 1). 16S rRNA gene sequences were clustered into OTUs at a similarity cutoff value of 97% using the UPARSE algorithm; OTUs were mapped to the SILVA Database to determine taxonomies. Abundances were recovered by mapping the demultiplexed reads to the UPARSE OTUs.

To infer the functional capabilities of the fecal microbiome based on the OTU (taxonomy) data, we used a bioinformatic approach, *Tax4Fun* [18]. This approach links the 16S rRNA gene sequences with the functional annotation of sequenced bacterial genomes by identifying a nearest neighbor based on a minimal 16S rRNA gene sequence similarity. Next, the predicted metagenomes were categorized by function at the Kyoto Encyclopedia of Genes and Genomes (KEGG) ortholog and pathway levels [19]. We tested for significant differences in the functional category abundances between cases and controls using Welch’s unequal variances t test. Resulting P values were adjusted for multiple hypothesis testing by converting to false discovery rate q values using the Benjamini–Hochberg procedure, with q values of <0.05 considered statistically significant. To validate the findings, we performed random permutation testing with 1000 permutations for each of the pathways of interest, which corresponds to the situation when the abundance of pathways is randomly assigned to cases and controls contained in dataset. Once the dataset was permuted, we tested for the differences in abundances between cases and controls. We repeated the randomization 1000 times and recorded the squared error of the models averaged for every repetition. Additionally, to further examine the differences in the pathways of interest, we constructed multivariable linear regression models adjusting for potential confounders (age, sex, race/ethnicity, maternal antibiotic use during pregnancy, history of prematurity, mode of delivery, feeding status, and lifetime history of antibiotic and corticosteroid use), based on a priori knowledge [5, 6, 9]. Furthermore, to determine the relationship between the abundance of bacteria genus and metabolic pathways of interest, we examined their correlations with the use of scatterplots fitting locally weighted scatterplot smoothed (LOWESS) curve as well as Spearman’s correlation. The analyses used R version 3.3 with *phyloseq* package [20] and STAMP version 2.1 [21].

## Results

At the four participating hospitals, a total of 40 infants hospitalized for bronchiolitis (cases) and 115 age-matched healthy infants (controls) were enrolled (Table 1). Overall, the median age was 3 months (IQR, 2–5 months) and 55% were male. All 155 fecal specimens had sufficient depth to obtain high degree of sequence coverage (rarefaction cutoff, 1470 reads/specimen; Fig. 1). The fecal microbiome were dominated by four genera: *Escherichia* (22%), *Bifidobacterium* (19%), *Enterobacter* (15%), and *Bacteroides* (13%). The characteristics of the fecal microbiome differed between cases and controls (Table 2). For example, infants with bronchiolitis had a higher proportion of *Bacteroides*-dominant profile and lower proportion of *Enterobacter/Veillonella*-dominant profile, compared to healthy controls (P = 0.01).

**Table 1:** Patient characteristics of 40 cases (infants with bronchiolitis) and 115 controls (healthy infants) at enrollment

Characteristics	Infants with bronchiolitis n = 40	Healthy control infants n = 115	P value*
Demographics			
Age (mo), median (IQR)	3.2 (1.6–4.9)	3.8 (2.0–4.9)	0.52
Male sex	22 (55)	64 (56)	0.99
Race/ethnicity			0.04
Non-hispanic white	23 (58)	61 (53)	
Non-hispanic black	6 (15)	11 (10)	
Hispanic	10 (25)	19 (17)	
Other	1 (3)	24 (21)	
Prenatal history			
Parental history of asthma	16 (40)	21 (18)	0.01
Maternal smoking during pregnancy	8 (20)	3 (3)	0.001
Maternal antibiotic use during pregnancy	11 (28)	13 (11)	0.02
Maternal antibiotic use during labor	12 (30)	35 (30)	0.82
Past medical history and home environmental characteristics			
Mode of birth, C-section	9 (23)	43 (37)	0.13
Prematurity (32–37 weeks)	12 (30)	11 (10)	0.004
Previous breathing problems before enrollment†	8 (20)	0 (0)	<0.001
History of eczema	8 (20)	17 (15)	0.56
Ever attended daycare	9 (23)	14 (12)	0.16
Smoking exposure at home	8 (20)	4 (3)	0.002
Mostly breastfed for the first 3 months of age	16 (40)	89 (77)	0.009
Systemic antibiotic use before enrollment^b^	8 (20)	13 (11)	0.24
Systemic corticosteroid use before enrollment	9 (23)	0 (0)	<0.001
Clinical course			
Systemic antibiotic use during pre-hospitalization visit	8 (20)	–	–
Systemic corticosteroid use during pre-hospitalization visit	3 (8)	–	–
Hospital length-of-stay (day), median (IQR)	3 (2–4)	–	–
Admission to intensive care unit	8 (20)	–	–
Use of mechanical ventilation^a^	5 (13)	–	–

Data are no. (%) of infants unless otherwise indicated. Percentages may not equal 100 because of missingness or rounding

*IQR* interquartile range

* Chi square, Fisher exact, or Wilcoxon-Mann–Whitney tests, as appropriate

^a^Defined as an infant having cough that wakes him/her at night and/or causes emesis, or when the child has wheezing or shortness of breath without cough

^b^Lifetime use of systemic antibiotic use before the enrollment. Infants with systemic antibiotic treatment in the preceding 7 days were not enrolled to the control group

**Table 2:** Richness, alpha-diversity, and relative abundance of fecal microbiome in infants with bronchiolitis and healthy controls

	Infants with bronchiolitis n = 40	Healthy control infants n = 115	P value
Richness, median (IQR)			
Number of genera	17 (13–23)	13 (10–18)	0.004
Alpha-diversity, median (IQR) shannon index	2.21 (1.68–2.65)	1.93 (1.44–2.49)	0.27
Relative abundance of 10 most abundant genera, mean (standard deviation)			
*Escherichia*	0.21 (0.24)	0.23 (0.26)	0.91*
*Bifidobacterium*	0.16 (0.20)	0.20 (0.21)	0.49*
*Enterobacter*	0.10 (0.21)	0.17 (0.24)	0.27*
*Bacteroides*	0.20 (0.23)	0.10 (0.19)	0.10*
*Veillonella*	0.03 (0.09)	0.06 (0.12)	0.31*
*Lachnospiraceae incertae sedis*	0.06 (0.10)	0.04 (0.10)	0.49*
*Streptococcus*	0.02 (0.09)	0.03 (0.05)	0.91*
*Clostridium* sensu *strictos 1*	0.01 (0.01)	0.03 (0.06)	0.16*
*Enterococcus*	0.01 (0.03)	0.02 (0.04)	0.48*
*Akkermansia*	0.02 (0.09)	0.02 (0.08)	0.91*
Microbiome profile, n (%)			0.01
*Bacteroides*-dominant profile	19 (48)	24 (21)	
*Bifidobacterium*-dominant profile	6 (15)	26 (23)	
*Escherichia*-dominant profile	10 (25)	36 (31)	
*Enterobacter/Veillonella*-dominant profile	5 (12)	29 (25)	

*IQR* interquartile range

* Benjamini–Hochberg corrected false discovery rate (q value) accounting for multiple comparisons

**Fig. 2:**
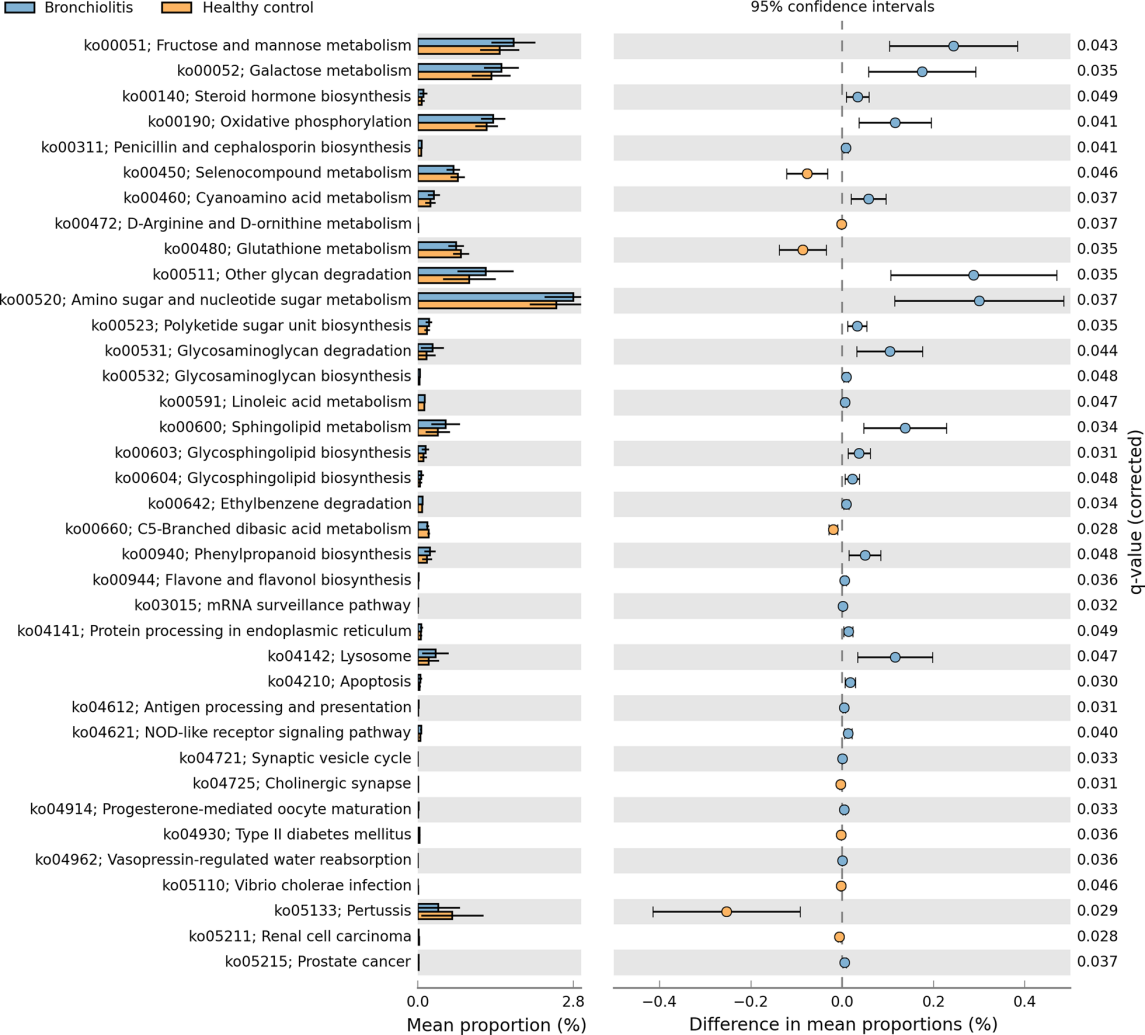
Predicted KEGG pathways with significant differences in relative abundance between infants with bronchiolitis and healthy controls. Of 274 KEGG pathways surveyed, the relative abundance of 37 genes was significantly different (false discovery rate, q < 0.05) between infants with bronchiolitis and healthy controls

**Fig. 3:**
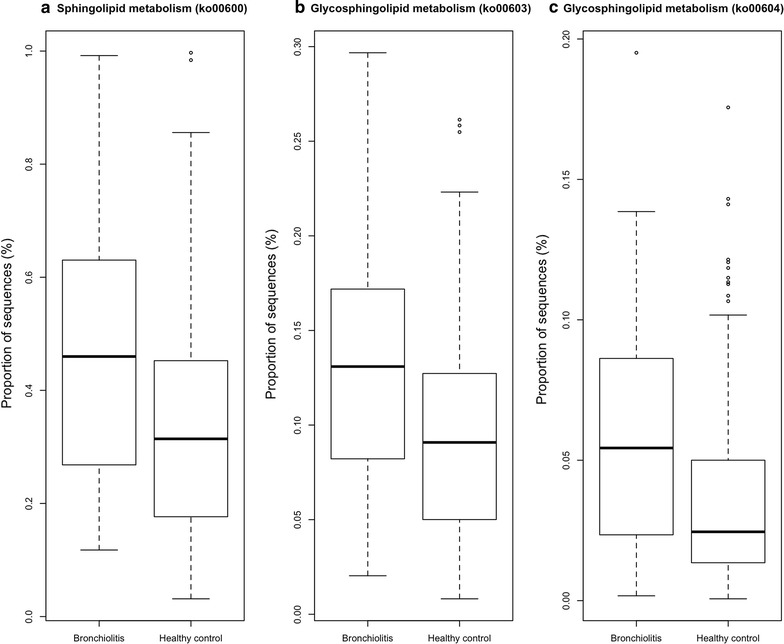
Box-whisker plots of the three sphingolipid metabolic pathways that distinguish between the fecal microbiome of infants with bronchiolitis and that of healthy controls. The predicted metagenome of fecal microbiome in infants with bronchiolitis had a higher abundance of the **a** ko00600 (q = 0.03), **b** ko00603 (q = 0.03), and **c** ko00604 (q = 0.048) pathways compared to that in healthy controls. The *horizontal line* represents the median; the *bottom* and the *top of the box* represent the 25th and the 75th percentiles; *whiskers* represent 5 and 95% percentiles

**Fig. 4:**
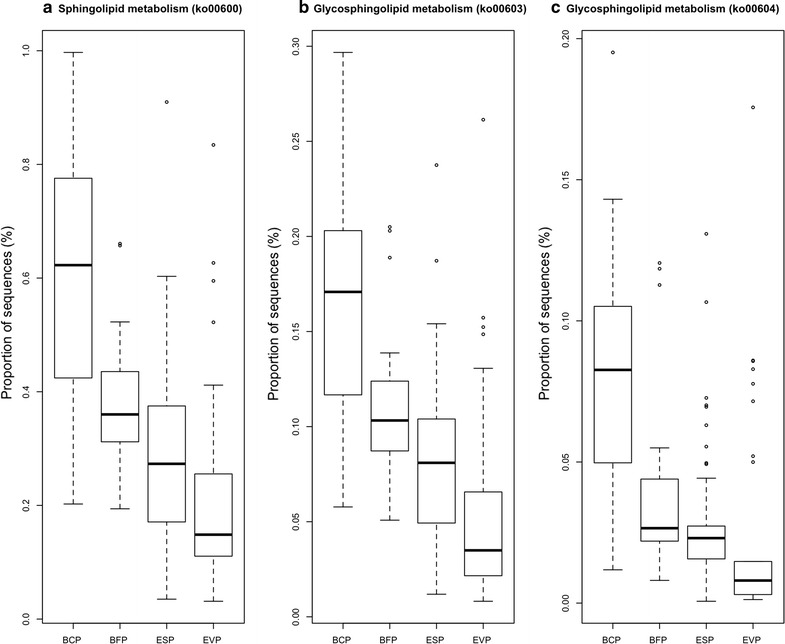
Box-whisker plots of the three sphingolipid metabolic pathways that distinguish four fecal microbiome profiles. The relative abundance of **a** ko00600, **b** ko00603, and **c** ko00604 pathways were consistently higher in infants with *Bacteroides*-dominant microbiome profile compared to the others (all q < 0.001). The four fecal microbiota profiles were derived using partitioning around medoids clustering method with Bray–Curtis distance. The optimal number of clusters was identified by the use of gap statistic. The *horizontal line* represents the median; the *bottom* and the *top of the box* represent the 25th and the 75th percentiles; *whiskers* represent 5 and 95% percentiles. *BCP Bacteroides*-dominant profile, *BFP Bifidobacterium*-dominant profile, *ESP Escherichia*-dominated profile, *EVP Enterobacter/Veillonella*-dominant profile

**Fig. 5:**
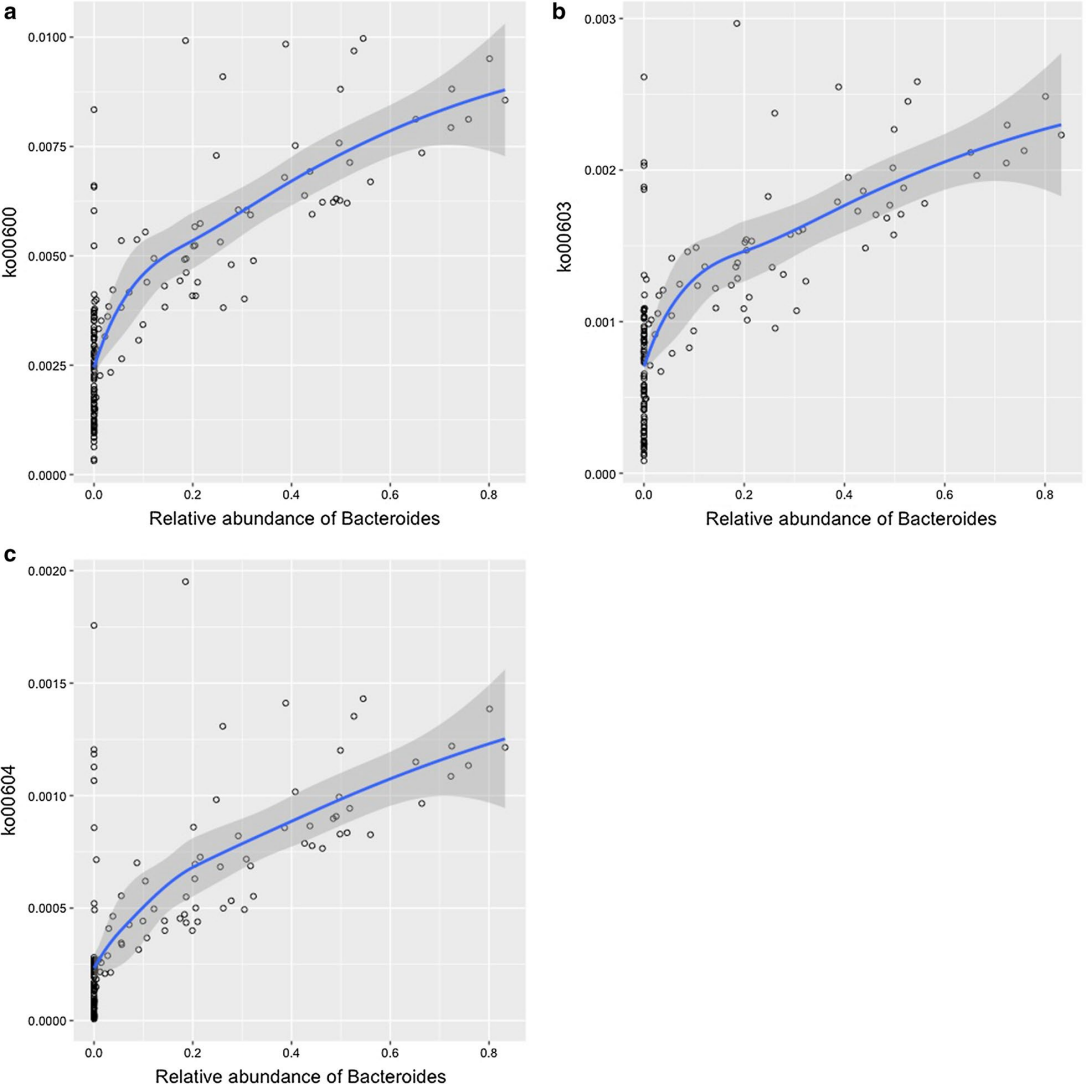
Correlations between the abundance of *Bacteroides* and the three sphingolipid metabolic pathways. There was a positive correlation between the abundance of *Bacteroides* and each of the three sphingolipid metabolic pathways. **a** ko00600 (Spearman’s *r* = 0.77; P < 0.001), **b** ko00603 (Spearman’s *r* = 0.73; P < 0.001), and **c** ko00604 (Spearman’s *r* = 0.74; P < 0.001). The *fitted line* represents locally weighted scatterplot smoothed (lowess) curve

Between the infants with bronchiolitis and healthy controls, we compared the functional potential of fecal microbiome inferred from the 16S rRNA gene sequencing data. Of 6402 KEGG orthologs (orthologous genes) surveyed, the abundances of 319 genes were significantly different (q < 0.05; Table 3). The functional differences involved genes with diverse metabolic functions—e.g., carbohydrate, amino acid, and lipid metabolism. To make the data presentation and interpretation more meaningful, the genes were further consolidated into 274 KEGG pathways. Among these, there were significant differences between bronchiolitis cases and healthy controls for 37 pathways, including lipid metabolic pathways (q < 0.05; Table 4; Fig. 2). Particularly, the fecal microbiome of bronchiolitis cases had *consistently* higher abundances of gene function related to the sphingolipid metabolic pathways compared to that of controls (all q < 0.05)—i.e., sphingolipid (ko00600) and glycosphingolipid (ko00603, ko00604) metabolic pathways (Fig. 3). For each of these 3 pathways, the permutation test was significant (all random permutation P < 0.05), supporting the validity of the observed between-group differences. In the multivariable models adjusting for 9 patient-level factors (age, sex, race/ethnicity, maternal antibiotic use during pregnancy, history of prematurity, mode of delivery, feeding status, and lifetime history of antibiotic and corticosteroid use), the difference in the abundances of 3 sphingolipid metabolic pathways remained significant (all P < 0.05). Additionally, these pathways were more abundant in infants with *Bacteroides*-dominant microbiome profile compared to the other microbiome profiles (all q < 0.001; Fig. 4). Likewise, there was a positive correlation between the abundance of *Bacteroides* genus and each of the 3 sphingolipid metabolic pathways (all P < 0.001; Fig. 5; Table 4).

**Table 3:** Predicted KEGG orthologs with significant differences in relative abundance between infants with bronchiolitis and healthy controls

KEGG orthologs	Mean abundance in cases (%)	Mean abundance in controls (%)	Raw P value	FDR corrected q value
K00179; indolepyruvate ferredoxin oxidoreductase, alpha subunit [EC:1.2.7.8]	0.020	0.009	<0.001	0.026
K00180; indolepyruvate ferredoxin oxidoreductase, beta subunit [EC:1.2.7.8]	0.007	0.003	<0.001	0.028
K02489; two-component system, cell cycle sensor kinase and response regulator [EC:2.7.13.3]	0.004	0.002	0.001	0.038
K03319; divalent anion:Na+ symporter, DASS family	0.019	0.044	<0.001	0.028
K08082; two-component system, LytT family, sensor histidine kinase AlgZ [EC:2.7.13.3]	0.011	0.005	0.001	0.041
K08196; MFS transporter, AAHS family, cis, cis-muconate transporter	0.001	0.002	0.002	0.044
K10715; two-component system, sensor histidine kinase RpfC [EC:2.7.13.3]	0.009	0.005	0.001	0.036
K10916; two-component system, CAI-1 autoinducer sensor kinase/phosphatase CqsS [EC:2.7.13.3 3.1.3.-]	0.001	0.000	0.001	0.031
K11382; MFS transporter, OPA family, phosphoglycerate transporter protein	0.006	0.015	0.000	0.028
K11383; two-component system, NtrC family, sensor histidine kinase KinB [EC:2.7.13.3]	0.001	0.000	0.001	0.034
K11520; two-component system, OmpR family, manganese sensing sensor histidine kinase [EC:2.7.13.3]	0.001	0.000	0.000	0.028
K11527; two-component system, unclassified family, sensor histidine kinase and response regulator [EC:2.7.13.3]	0.023	0.011	0.001	0.038
K15850; two-component system, autoinducer 1 sensor kinase/phosphatase LuxN [EC:2.7.13.3 3.1.3.-]	0.001	0.000	<0.001	0.026
K15913; UDP-4-amino-4,6-dideoxy-*N*-acetyl-d-glucosamine 4-acetyltransferase [EC:2.3.1.-]	0.000	0.000	0.002	0.049
K16014; ATP-binding cassette, subfamily C, bacterial CydCD	0.005	0.010	0.002	0.042
K00176; 2-oxoglutarate ferredoxin oxidoreductase subunit delta [EC:1.2.7.3]	0.001	0.001	0.001	0.035
K00177; 2-oxoglutarate ferredoxin oxidoreductase subunit gamma [EC:1.2.7.3]	0.010	0.005	0.001	0.041
K00200; formylmethanofuran dehydrogenase subunit A [EC:1.2.99.5]	0.001	0.000	0.001	0.038
K00316; spermidine dehydrogenase [EC:1.5.99.6]	0.000	0.000	0.002	0.046
K00406; cytochrome c oxidase cbb3-type subunit III	0.002	0.004	0.001	0.030
K00436; hydrogen dehydrogenase [EC:1.12.1.2]	0.002	0.001	<0.001	0.023
K00824; d-alanine transaminase [EC:2.6.1.21]	0.004	0.008	0.002	0.045
K00832; aromatic-amino-acid transaminase [EC:2.6.1.57]	0.010	0.020	<0.001	0.026
K00856; adenosine kinase [EC:2.7.1.20]	0.002	0.001	0.001	0.034
K00908; Ca2+/calmodulin-dependent protein kinase [EC:2.7.11.17]	0.001	0.000	<0.001	0.023
K01235; alpha-glucuronidase [EC:3.2.1.139]	0.017	0.008	0.002	0.041
K01601; ribulose-bisphosphate carboxylase large chain [EC:4.1.1.39]	0.003	0.010	0.002	0.042
K01841; phosphoenolpyruvate phosphomutase [EC:5.4.2.9]	0.008	0.004	0.002	0.049
K01906; 6-carboxyhexanoate–CoA ligase [EC:6.2.1.14]	0.003	0.007	0.001	0.037
K01912; phenylacetate-CoA ligase [EC:6.2.1.30]	0.037	0.018	0.001	0.041
K02121; V-type H+ -transporting ATPase subunit E [EC:3.6.3.14]	0.010	0.005	<0.001	0.024
K02655; type IV pilus assembly protein PilE	0.002	0.005	<0.001	0.023
K03330; glutamyl-tRNA (Gln) amidotransferase subunit E [EC:6.3.5.7]	0.001	0.003	0.001	0.042
K03404; magnesium chelatase subunit D [EC:6.6.1.1]	0.007	0.019	0.002	0.046
K03756; putrescine:ornithine antiporter	0.007	0.016	<0.001	0.024
K04561; nitric oxide reductase subunit B [EC:1.7.2.5]	0.006	0.017	<0.001	0.026
K05586; bidirectional [NiFe] hydrogenase diaphorase subunit [EC:1.6.5.3]	0.001	0.000	0.001	0.032
K05588; bidirectional [NiFe] hydrogenase diaphorase subunit [EC:1.6.5.3]	0.001	0.000	0.002	0.043
K05589; cell division protein FtsB	0.002	0.003	<0.001	0.024
K05989; alpha-l-rhamnosidase [EC:3.2.1.40]	0.056	0.028	0.002	0.045
K06138; pyrroloquinoline quinone biosynthesis protein D	0.001	0.000	0.001	0.034
K07326; hemolysin activation/secretion protein	0.001	0.003	<0.001	0.026
K07536; 2-ketocyclohexanecarboxyl-CoA hydrolase [EC:3.1.2.-]	0.001	0.002	0.001	0.041
K09002; hypothetical protein	0.006	0.018	0.001	0.041
K09020; ureidoacrylate peracid hydrolase [EC:3.5.1.110]	0.002	0.003	0.001	0.031
K09162; hypothetical protein	0.003	0.008	0.001	0.034
K09459; phosphonopyruvate decarboxylase [EC:4.1.1.82]	0.005	0.003	0.003	0.050
K09477; citrate:succinate antiporter	0.008	0.016	0.001	0.034
K09758; aspartate 4-decarboxylase [EC:4.1.1.12]	0.015	0.007	<0.001	0.026
K09800; hypothetical protein	0.025	0.053	<0.001	0.026
K09824; hypothetical protein	0.007	0.014	<0.001	0.026
K10960; geranylgeranyl reductase [EC:1.3.1.83]	0.001	0.000	<0.001	0.027
K10974; cytosine permease	0.007	0.016	<0.001	0.026
K11016; hemolysin	0.001	0.002	0.001	0.029
K11106; l-tartrate/succinate antiporter	0.009	0.020	<0.001	0.026
K11607; manganese/iron transport system ATP-binding protein	0.004	0.009	<0.001	0.022
K11707; manganese/zinc/iron transport system substrate-binding protein	0.003	0.007	0.001	0.041
K11708; manganese/zinc/iron transport system permease protein	0.003	0.007	0.002	0.042
K11709; manganese/zinc/iron transport system permease protein	0.004	0.008	0.001	0.034
K11719; lipopolysaccharide export system protein LptC	0.003	0.007	<0.001	0.023
K11931; biofilm PGA synthesis lipoprotein PgaB [EC:3.-.-.-]	0.008	0.016	<0.001	0.028
K12341; adhesin YadA	0.005	0.012	0.001	0.041
K12681; pertactin	0.001	0.002	0.002	0.041
K12982; heptosyltransferase I [EC:2.4.-.-]	0.001	0.002	<0.001	0.028
K13256; protein PsiE	0.003	0.006	<0.001	0.029
K13498; indole-3-glycerol phosphate synthase/phosphoribosylanthranilate isomerase [EC:4.1.1.48 5.3.1.24]	0.008	0.017	<0.001	0.022
K13818; molybdopterin-guanine dinucleotide biosynthesis protein	0.001	0.003	0.001	0.029
K14448; (2S)-methylsuccinyl-CoA dehydrogenase	0.002	0.001	0.001	0.032
K14564; nucleolar protein 56	0.001	0.000	0.002	0.043
K14665; amidohydrolase [EC:3.5.1.-]	0.002	0.004	0.001	0.031
K15125; filamentous hemagglutinin	0.032	0.092	0.000	0.024
K15669; d-glycero-alpha-d-manno-heptose 1-phosphate guanylyltransferase [EC:2.7.7.71]	0.002	0.001	0.001	0.031
K15905; nitrite oxidoreductase alpha subunit	0.001	0.003	0.001	0.041
K16201; dipeptide transport system permease protein	0.001	0.003	0.002	0.045

Of 6402 KEGG orthologs surveyed, the relative abundances of 319 genes were significantly different (FDR, q < 0.05) between infants with bronchiolitis and healthy controls. Of these, 74 orthologs with a ratio of abundance >2.0 are displayed

**Table 4:** Predicted KEGG pathways with significant differences in relative abundance between infants with bronchiolitis and healthy controls

KEGG pathway	Difference in relative abundance				Correlation with *Bacteroides* abundance	
	Mean abundance in cases (%)	Mean abundance in controls (%)	Raw P value	FDR corrected q value	Spearman’s *rho*	P value
ko00051; fructose and mannose metabolism	1.691	1.447	0.001	0.043	0.55	<0.001
ko00052; galactose metabolism	1.473	1.298	0.004	0.035	0.66	<0.001
ko00140; steroid hormone biosynthesis	0.098	0.063	0.007	0.049	0.66	<0.001
ko00190; oxidative phosphorylation	1.332	1.215	0.005	0.041	0.41	<0.001
ko00311; penicillin and cephalosporin biosynthesis	0.068	0.060	0.001	0.041	0.44	<0.001
ko00450; selenocompound metabolism	0.626	0.703	0.001	0.046	−0.61	<0.001
ko00460; cyanoamino acid metabolism	0.280	0.222	0.004	0.037	0.82	<0.001
ko00472; d-arginine and d-ornithine metabolism	0.001	0.002	0.004	0.037	−0.52	<0.001
ko00480; glutathione metabolism	0.678	0.764	0.001	0.035	−0.49	<0.001
ko00511; other glycan degradation	1.198	0.910	0.002	0.035	0.72	<0.001
ko00520; amino sugar and nucleotide sugar metabolism	2.751	2.450	0.002	0.037	0.77	<0.001
ko00523; polyketide sugar unit biosynthesis	0.196	0.163	0.002	0.035	0.80	<0.001
ko00531; glycosaminoglycan degradation	0.256	0.152	0.005	0.044	0.73	<0.001
ko00532; glycosaminoglycan biosynthesis	0.035	0.026	0.001	0.048	0.72	<0.001
ko00591; linoleic acid metabolism	0.120	0.113	0.006	0.047	0.52	<0.001
*ko00600; sphingolipid metabolism*	*0.489*	*0.351*	*0.003*	*0.034*	*0.77*	*<0.001*
*ko00603; glycosphingolipid biosynthesis*	*0.134*	*0.097*	*0.003*	*0.031*	*0.73*	*<0.001*
*ko00604; glycosphingolipid biosynthesis*	*0.061*	*0.039*	*0.006*	*0.048*	*0.74*	*<0.001*
ko00642; Ethylbenzene degradation	0.084	0.075	0.002	0.034	0.25	0.001
ko00660; C5-branched dibasic acid metabolism	0.171	0.191	<0.001	0.028	−0.50	<0.001
ko00940; phenylpropanoid biosynthesis	0.213	0.163	0.006	0.048	0.81	<0.001
ko00944; flavone and flavonol biosynthesis	0.015	0.009	0.002	0.036	0.77	<0.001
ko03015; mRNA surveillance pathway	0.003	0.001	0.003	0.032	0.80	<0.001
ko04141; protein processing in endoplasmic reticulum	0.063	0.049	0.006	0.049	0.69	<0.001
ko04142; lysosome	0.310	0.194	0.006	0.047	0.76	<0.001
ko04210; apoptosis	0.043	0.025	0.002	0.030	0.69	<0.001
ko04612; antigen processing and presentation	0.014	0.010	0.002	0.031	0.58	<0.001
ko04621; NOD-like receptor signaling pathway	0.056	0.042	0.001	0.040	0.74	<0.001
ko04721; synaptic vesicle cycle	0.001	0.000	0.002	0.033	0.58	<0.001
ko04725; cholinergic synapse	0.002	0.005	0.003	0.031	−0.12	0.14
ko04914; progesterone-mediated oocyte maturation	0.014	0.010	0.002	0.033	0.58	<0.001
ko04930; type II diabetes mellitus	0.026	0.028	0.001	0.036	−0.47	<0.001
ko04962; vasopressin-regulated water reabsorption	0.001	0.000	0.002	0.036	0.58	<0.001
ko05110; vibrio cholerae infection	0.001	0.004	0.006	0.046	−0.41	<0.001
ko05133; pertussis	0.356	0.609	0.002	0.029	−0.24	0.03
ko05211; renal cell carcinoma	0.013	0.019	<0.001	0.028	−0.58	<0.001
ko05215; prostate cancer	0.016	0.011	0.002	0.037	0.63	<0.001

Italics results are the pathways of interest (sphingolipid metabolic pathways)

## Discussion

By predicting the functional potential of the fecal microbiome from 40 infants with bronchiolitis and 115 healthy age-matched controls enrolled in a case–control study, we found significant differences in the abundance of genes related to multiple metabolic pathways. Of these, the gene function related to sphingolipid metabolic pathways was *consistently* more abundant in the fecal microbiome of bronchiolitis cases compared to that of healthy controls. The current study extends the previously identified association of *Bacteroides*-dominated fecal microbiome profile with higher likelihoods of bronchiolitis by demonstrating the functional potential of the gut microbiome in infants.

Sphingolipids are a class of complex lipids containing a backbone of sphingoid bases. These lipids have long been known as structural components of human cell membranes and as a component of surfactant, but have more recently emerged as signaling molecules that modulate the host immune response and contribute to the pathogenesis of respiratory diseases, such as bronchiolitis, pneumonia, and asthma [7, 22]. While sphingolipids production is ubiquitous in eukaryotes, it is also produced by several bacteria genera such as *Bacteroides*, *Prevotella*, and *Porphyromonas* [12]. Recently, experimental models reported that *Bacteroides*-derived sphingolipids (e.g., α-galactosylceramide) play an important role in host immunomodulation similar to lipopolysaccharide (LPS), another family of bacteria-derived glycolipid. For example, Wieland Brown et al. demonstrated that *Bacteroides*-derived α-galactosylceramide binds to CD1d and activates mouse and human invariant natural killer T (iNKT) cells both in vitro and in vivo [12]. In contrast, An et al., using neonatal mouse models, found that treatment with a different *Bacteroides*-derived glycosphingolipids (GSL-Bf717) reduces the number of colonic iNKT cells and subsequent colonic inflammation [13]. Although reverse causation—e.g., bronchiolitis *per se* or treatments for bronchiolitis result in perturbation of the fecal microbiome—is also possible, these prior studies, coupled with our findings, collectively suggest that *Bacteroides*-dominant microbiome in the gut, through their sphingolipid production, may contribute to inappropriate immune responses and bronchiolitis pathogenesis in infants. Our data should encourage future investigations into the mechanisms linking the individual gut microbiome-derived metabolites to the host immune response in the gut and respiratory tract (the gut-lung axis).

In sum, on the basis of the predicted metagenome in this case–control study, we found significant differences in the functional potential of fecal microbiome between infants with bronchiolitis and healthy controls. Particularly, the fecal microbiome in infants with bronchiolitis had consistently higher abundances of gene function related to the sphingolipid metabolic pathways. Although causal inferences remain premature, our data may suggest a potential link between the bacteria-derived metabolites, modulations of host immune response, and development of bronchiolitis. Our findings should facilitate further metagenomic, metatranscriptomic, and metabolomic (including *Bacteroides*-derived galactosylceramide [13]) investigations into the role of gut microbiome in the bronchiolitis pathogenesis. Our data also encourage researchers to integrate these “omics” approaches with mechanistic evaluations in experimental models in order to develop new preventive and therapeutic strategies (e.g., microbiome modification) for infants with bronchiolitis.

## Limitations

Our study has several potential limitations. First, the location of fecal sample collection differed between cases and controls. However, in both populations, the fecal samples were refrigerated immediately after collection and the literature reported that refrigeration is associated with no significant alteration in fecal microbiota composition [23]. Second, the functional potential of fecal microbiome was inferred from the 16S rRNA gene sequencing data rather than measured by metabolomics or metatranscriptomics, or from metagenomic sequencing. However, a study has shown a strong correlation between the predicted metagenome and metagenome sequencing data (*r* > 0.85) in the NIH Human Microbiome Project samples (including fecal samples) [18]. Third, the concentration of metabolites was not measured in the fecal samples. This is an important area for examination in our future work. Lastly, the study design precluded us from examining the succession of fecal microbiome and its relation to the development of respiratory disease in early childhood. To address this question, the study populations are currently being followed longitudinally to age 6 years, with fecal sample collections at multiple time-points.

## Abbreviations

iNKT: invariant natural killer T
KEGG: Kyoto Encyclopedia of Genes and Genomes
MARC: Multicenter Airway Research Collaboration
OTU: operational taxonomic unit
